# Reciprocal Regulation as a Source of Ultrasensitivity in Two-Component Systems with a Bifunctional Sensor Kinase

**DOI:** 10.1371/journal.pcbi.1003614

**Published:** 2014-05-08

**Authors:** Ronny Straube

**Affiliations:** Analysis and Redesign of Biological Networks Group, Max Planck Institute for Dynamics of Complex Technical Systems, Magdeburg, Germany; ENS de Lyon, France

## Abstract

Two-component signal transduction systems, where the phosphorylation state of a regulator protein is modulated by a sensor kinase, are common in bacteria and other microbes. In many of these systems, the sensor kinase is bifunctional catalyzing both, the phosphorylation and the dephosphorylation of the regulator protein in response to input signals. Previous studies have shown that systems with a bifunctional enzyme can adjust the phosphorylation level of the regulator protein independently of the total protein concentrations – a property known as concentration robustness. Here, I argue that two-component systems with a bifunctional enzyme may also exhibit ultrasensitivity if the input signal reciprocally affects multiple activities of the sensor kinase. To this end, I consider the case where an allosteric effector inhibits autophosphorylation and, concomitantly, activates the enzyme's phosphatase activity, as observed experimentally in the PhoQ/PhoP and NRII/NRI systems. A theoretical analysis reveals two operating regimes under steady state conditions depending on the effector affinity: If the affinity is low the system produces a graded response with respect to input signals and exhibits stimulus-dependent concentration robustness – consistent with previous experiments. In contrast, a high-affinity effector may generate ultrasensitivity by a similar mechanism as phosphorylation-dephosphorylation cycles with distinct converter enzymes. The occurrence of ultrasensitivity requires saturation of the sensor kinase's phosphatase activity, but is restricted to low effector concentrations, which suggests that this mode of operation might be employed for the detection and amplification of low abundant input signals. Interestingly, the same mechanism also applies to covalent modification cycles with a bifunctional converter enzyme, which suggests that reciprocal regulation, as a mechanism to generate ultrasensitivity, is not restricted to two-component systems, but may apply more generally to bifunctional enzyme systems.

## Author Summary

Bacteria often use two-component systems to sense and respond to environmental changes, which involves autophosphorylation of a sensor kinase and phosphotransfer to a cognate response regulator. However, despite conservation of this ‘classical’ scheme there exist substantial variations in the mechanism of phosphotransfer among systems. Also, many sensor kinases exhibit phosphatase activity raising the question whether such a bifunctional architecture enables special regulatory properties in the response behavior to input signals. According to previous studies, classical two-component systems are unlikely to produce sigmoidal response curves (ultrasensitivity) if the sensor protein is bifunctional. Here, I argue that this is not necessarily true if the input stimulus (allosteric effector) reciprocally affects multiple activities of the sensor kinase, as it seems to be common for bifunctional enzymes. To this end, I propose and analyze an extension of the experimentally well-supported Batchelor-Goulian model which shows that ultrasensitivity requires a high-affinity effector and saturation of the phosphatase activity. The underlying mechanism involves sequestration of the effector by the sensor kinase which restricts the occurrence of ultrasensitivity to sufficiently low effector concentrations. Hence, this operating regime might be useful to sense effector limitations or to amplify weak input signals.

## Introduction

**Figure 1:**
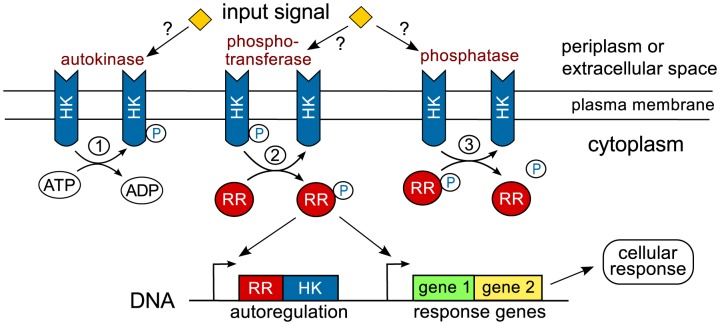
Signal flow in classical two-component systems. Typically, the sensor histidine kinase (HK) is a (dimeric) transmembrane protein which senses extracellular signals directly or through their concentration in the periplasm [3]. In some case, signal-sensing may also occur in the cytosol or in the plasma membrane [43]. The HK exhibits up to three distinct activities: (1) autokinase activity leading to the autophosphorylation of the HK, (2) phosphotransfer to the response regulator (RR) and (3) phosphatase activity towards the phosphorylated form of the RR (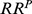). In general, the input signal may affect all three HK activities although autokinase and phosphatase activities appear to be the most common targets of regulation [20], [21], [44], [45]. The phosphorylated form of the response regulator often acts as a transcription factor which activates or represses a particular set of response genes including those of the RR and the HK themselves (autoregulation).

Two-component systems (TCSs) are modular signal transduction systems which are utilized by bacteria and other microbes to respond to intracellular or environmental stimuli [1], [2]. ‘Classical’ TCSs consist of a sensor histidine kinase (HK) and a cognate response regulator (RR), which often acts as a transcription factor to activate or repress a particular set of response genes. Upon stimulation, the HK autophosphorylates at a conserved histidine residue and transfers the phosphoryl group to an aspartate residue in the receiver domain of the RR. Often, the unphosphorylated form of the HK also exhibits phosphatase activity towards the phosphorylated form of the RR (RR-P) endowing many HKs with a bifunctional design (Fig. 1). In addition, some RRs exhibit intrinsic phosphatase activity which leads to autodephosphorylation of RR-P with a half-life ranging between seconds to hours [1].

Even though the overall signal flow from the sensor kinase to the response regulator is well-conserved between different systems there exist substantial variations in the particular mechanism through which the phosphoryl group is transferred to the regulator protein [3]. To better understand their regulatory properties it has become a useful strategy to compare different TCS architectures based on their potential input-output behavior. Following that strategy, it has been argued that phosphorelay systems, where the phosphotransfer to the RR does not occur in a single step but via additional intra- or intermolecular reactions [4], may generate ultrasensitivity and robustness against noise [5]. Systems with a split histidine kinase comprise another class of TCSs where a functional HK is generated through binary association between two distinct proteins each of which alone would not be able to phosphorylate the cognate RR(s) [6]. A theoretical study suggested that such systems can potentially exhibit ultrasensitivity and bistability if the phosphatase activity is predominantly located on the free form of one of the proteins making up the split kinase [7]. Yet another study compared TCSs with a mono- and a bifunctional HK arriving at the conclusion that ultrasensitivity and bistability can also occur in classical TCSs if the unphosphorylated forms of the HK and the RR form a dead-end complex and if the dephosphorylation of the RR mainly occurs via an HK-independent phosphatase [8].

In contrast, systems with a bifunctional design are expected to generate graded responses to input stimuli [8]–[10] and to mediate concentration robustness [11]–[13]. The latter property means that the system response (concentration of phosphorylated RR) is invariant with respect to variations of the total RR and HK concentrations, at least in a certain range of concentrations. Moreover, based on theoretical studies of covalent modification cycles with a bifunctional converter enzyme it has been argued that ultrasensitivity is unlikely to occur in such systems if the bifunctional enzyme employs only a single catalytic site for its opposing activities [14], [15]. Based on this argument it, thus, appears unlikely that classical two-component systems with a bifunctional sensor kinase would exhibit ultrasensitivity given that the phosphotransferase and phosphatase activities of the sensor kinase are believed to occur on a single catalytic site in the dimerization domain of the protein [16], [17]. Interestingly, this conclusion does not apply to bifunctional enzymes with two distinct catalytic sites where ultrasensitivity may arise from the formation of a ternary complex between the enzyme and its two substrates [18] as observed experimentally in the uridylylation cycle of the PII protein [19].

**Figure 2:**
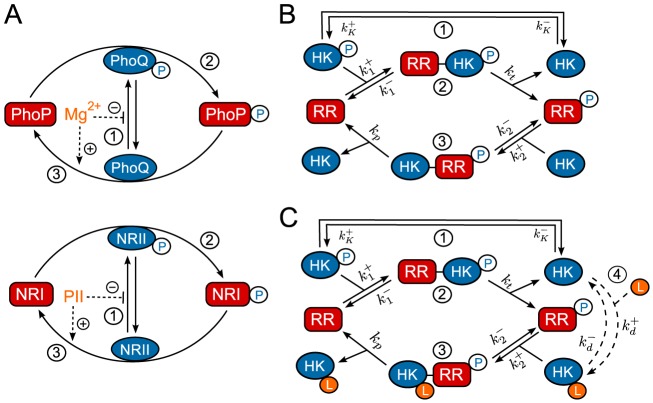
Reciprocal regulation in two-component systems. (A) Schematic representation of reciprocal regulation in the PhoQ/PhoP [20] and NRII/NRI systems [21]. In both cases, an allosteric effector (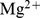 or PII) inhibits autophosphorylation of the sensor kinase and increases the enzyme's phosphatase activity. (B) Batchelor-Goulian model [11] based on the three activities of the sensor kinase (cf. Fig. 1): (1) Autophosphorylation of the sensor kinase (HK), (2) phosphotransfer to the response regulator (RR) and (3) dephosphorylation of the RR. Cofactors such as ATP are assumed to be constant. (C) Extension of the Batchelor-Goulian model to include reciprocal regulation of the HK's activities as schematized in (A). Binding of the allosteric effector 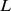 (4) inhibits autophosphorylation (1) and activates the phosphatase activity (3) of the sensor kinase. For simplicity, the free form of the enzyme (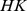) is assumed to have no phosphatase activity whereas the effector-bound form (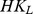) is assumed to have no autokinase activity.

In the present study, I wish to argue that ultrasensitivity may still occur in two-component systems with a bifunctional enzyme kinase if the input signal reciprocally affects multiple activities of the sensor kinase. Reciprocal regulatory patterns have been observed in the PhoQ/PhoP system which mediates adaption in response to 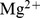 limitation as well as in the NRII/NRI system which mediates adaptation to nitrogen limitation by sensing the concentration of deuridylylated PII protein in the cytosol. In both cases, binding of an allosteric effector (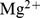 or PII) inhibits the autokinase activity and, concomitantly, activates the phosphatase activity of the respective sensor protein (Fig. 2A) [20], [21]. Indeed, based on structural analysis of HK domains it has been argued that reciprocal regulation could be quite common for bifunctional enzymes [17].

In a first step, the impact of reciprocal regulation is analyzed in covalent modification cycles with a bifunctional converter enzyme, which will serve as a ‘toy’ model that allows for an intuitive understanding of the potential mechanism for the generation of ultrasensitivity. In a second step, it will be shown that the same mechanism may also generate ultrasensitivity in classical TCSs with a bifunctional sensor kinase. To this end, an extension of the experimentally well-supported Batchelor-Goulian model (see below) is proposed which assumes that autokinase and phosphatase activities of the HK are reciprocally regulated by an allosteric effector (Fig. 2C). Analysis of this model shows that if the affinity of the effector is low (as in the case of 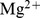 for PhoQ) the system exhibits a graded response to changes in the effector concentration and stimulus-dependent concentration robustness – in agreement with experiments in the PhoQ/PhoP system [22]. In contrast, a high-affinity effector may lead to ultrasensitivity at low effector concentrations, but requires saturation of the sensor kinase's phosphatase activity. Comparison of the model predictions with *in vitro* experiments suggests that in the NRII/NRI system the occurrence of ultrasensitivity is (partly) suppressed by the intrinsic autophosphatase activity of NRI.

### Concentration robustness in the Batchelor-Goulian model

To rationalize the occurrence of concentration robustness in the EnvZ/OmpR system of *E. coli*, Batchelor and Goulian proposed a simple mathematical model based on the three activities of the bifunctional EnvZ (denoted by HK in Fig. 2B). Guided by the observation that the total OmpR concentration is much larger than that of EnvZ [23] (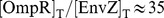) they have argued that, in the limit 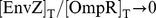, the steady state concentration of OmpR-P (denoted by 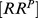 in Fig. 2B) is determined by a quadratic equation [11], which can be written in the form (*SI Text S1*)
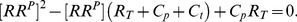
(1)Here, 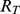 denotes the total OmpR concentration, and the parameters 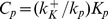 and 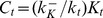 are proportional to the Michaelis-Menten constants associated with the phosphatase (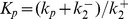) and phosphotransferase (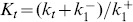) reactions. Note that Eq. (1) does not depend on the total EnvZ concentration (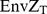). Hence, the Batchelor-Goulian model predicts that, in the limit 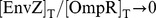, the concentration of OmpR-P is approximately independent of variations in the total concentration of the sensor kinase, i.e. [OmpR-P] exhibits (concentration) robustness with respect to changes in 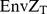.

Interestingly, Eq. (1) also predicts concentration robustness of 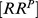 with respect to the total concentration of the response regulator (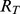) under certain conditions. To see this more explicitly, it is worth mentioning that a structurally similar equation has been analyzed previously in the context of concentration robustness for covalent modification cycles with a bifunctional converter enzyme [24]. This analysis has shown that the shape of the stimulus-response curve, described by Eq. (1), depends on the relative magnitude between the two parameters 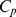 and 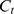 [18]. To this end, it is useful to consider two limiting cases corresponding to 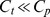 and 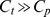. In the first case, the physiologically reasonable solution of Eq. (1) can be approximated by (*SI Text S1*)
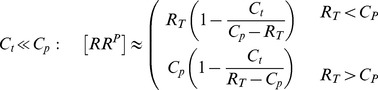
(2)whereas, in the second case, one obtains the approximate solution
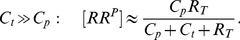
(3)In any case, from the expressions in Eqs. (2) and (3) it is readily apparent that 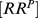 becomes independent of the total RR concentration if the latter is sufficiently large, i.e. 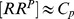 if 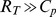 (Eq. 2) or 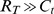 (Eq. 3). Hence, if 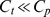, the parameter 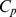 determines both, the threshold concentration beyond which 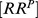 becomes approximately constant as well as the value of that constant. In contrast, if 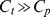, the predicted threshold concentration (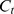) is much larger than the asymptotic phosphorylation level of the response regulator (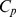). Also, the approach to the asymptotic level is different for the two regimes: If 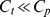, 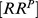 increases approximately linearly with 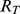 up to the threshold (Eq. 2) whereas, in the opposite case, it increases hyperbolically (Eq. 3). Due to the linear relationship between 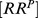 and 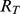 in Eq. (2) the regime 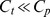 has been called ‘signal-transducing’ in Ref. [25].

Together, Eqs. (2) and (3) suggest that there exist two different regimes for the occurrence of concentration robustness and, as will be shown below, there is experimental evidence for either case.

### Experimental support for the Batchelor-Goulian model

**Figure 3:**
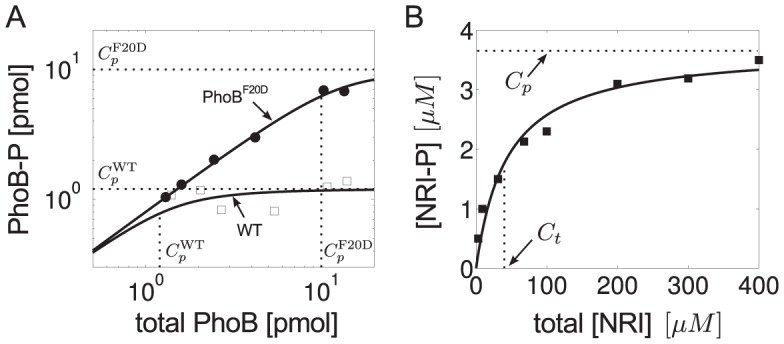
Experimental observations of concentration robustness in TCSs. Comparison between predictions of the Batchelor-Goulian model and measurements in the PhoR/PhoB [26] and NRII/NRI systems [27]. (A) Symbols denote measurements of PhoB-P as a function of total PhoB amounts in the wild-type system (open squares) and in a 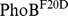 mutant strain (filled circles) (data were taken from Fig. 4C in Ref. [26]). Solid lines were calculated from Eq. (31) with 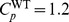 pmol, 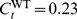 pmol and 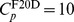 pmol, 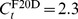 pmol. Note that 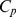 (dotted lines) determines both, the threshold amount of total PhoB beyond which PhoB-P becomes constant as well as the value of that constant, as expected from Eq. (2). (B) Symbols denote *in vitro* measurements of NRI-P as a function of total NRI (reproduced from Fig. 4A in Ref. [27]). Solid line represents the best fit of the data to Eq. (3) with 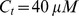 and 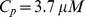, which indicates that the NRII/NRI system operates in the regime 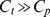.

To test the predictions of their model, Batchelor and Goulian measured changes in the transcriptional activity of OmpR-controlled genes using a two-fluorescent reporter strain, which provided indirect evidence for concentration robustness of OmpR-P. Recently, Gao and Stock directly confirmed the predictions of the Batchelor-Goulian model in the PhoR/PhoB system using a Phos-tag based method allowing for a quantification of the PhoB-P levels as a function of total PhoB amounts [26]. Experiments were performed with the wild-type (WT) system as well as with a PhoB mutant (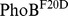) which exhibits reduced interaction strength (affinity) with PhoR. Both measurements could be well described by Eq. (1) with a 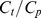 ratio varying between 0.1–0.2 (Fig. 3A, solid lines). Overlaying the response curves with the respective 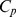 values (dotted lines) indicates that the PhoR/PhoB system operates in the regime 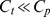 since the threshold concentration (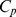), beyond which PhoB-P becomes constant, is approximately equal to the value of that constant, as expected from Eq. (2). The observed shift of the threshold concentration in the mutant strain results from the reduced affinity of 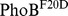 which is associated with a larger value for 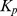. Since 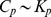, increasing 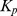 leads to an increased value of 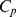 so that the asymptotically constant phosphorylation level of 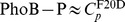 is reached at higher total PhoB concentrations, i.e. for total 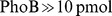 (Fig. 3A).

Concentration robustness has also been observed in the reconstituted NRII/NRI system of *E. coli* under *in vitro* conditions [27]. However, in that case the shape of the response curve is quite different (Fig. 3B): The dependence between [NRI-P] and total [NRI] does not appear to be linear below the threshold concentration and the asymptotically constant phosphorylation level (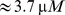) is only reached for very large values of total [NRI] (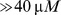). Together, this indicates that the NRII/NRI system operates in the regime 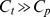 and, indeed, fitting the measurement data to Eq. (3) supports this view (Fig. 3B, solid line). Moreover, since *in vivo* concentrations of NRI are typically much lower than the threshold concentration of 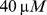 [28] it has been argued that, in the NRII/NRI system, concentration robustness will most likely not play a role under physiological conditions [27].

## Results

### Ultrasensitivity in covalent modification cycles with a bifunctional enzyme

**Figure 4:**
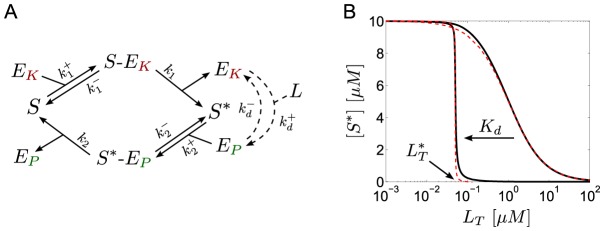
Ultrasensitivity in covalent modification cycles with a bifunctional converter enzyme. (A) Reaction scheme: A substrate molecule (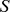) undergoes reversible phosphorylation by a bifunctional converter enzyme which can exist in two activity states. Binding of the allosteric effector 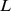 inhibits the kinase activity (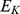) and, concomitantly, activates the phosphatase activity (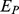) of the enzyme. (B) As the value of the dissociation constant 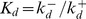 is lowered from 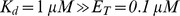 to 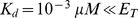 (from right to left) the steady state curve becomes ultrasensitive near the transition point 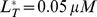, as defined in Eq. (21). The solid lines were computed from the full model using Eqs. (4)–(7). Dashed lines were computed from the reduced models using Eq. (14) (right curve) and Eq. (18) (left curve). Parameters: 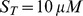, 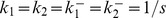, 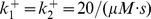 so that 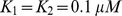, 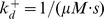 and 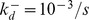 (for 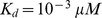) or 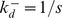 (for 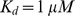).

To understand how ultrasensitivity may arise in TCSs with a bifunctional HK it will be helpful to analyze the consequences of reciprocal regulation in a related, but more simple system first. To this end, the reaction mechanism in Fig. 4A, which describes the reversible phosphorylation of a substrate 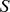 by a bifunctional enzyme 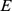, is considered. The enzyme exhibits both, kinase (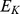) and phosphatase (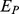) activities, which catalyze the phosphorylation (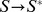) and dephosphorylation reactions (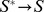), respectively. The transition between the two activity states is mediated through binding of an allosteric effector 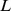. For simplicity, it is assumed that 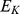 has no phosphatase activity and, conversely, 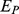 has no kinase activity so that effector-binding effectively inhibits the enzyme's kinase activity and, concomitantly, activates its phosphatase activity. Note that this system is similar to TCSs with a bifunctional sensor kinase where the autophosphorylation and phosphotransfer reactions are replaced by a covalent modification (cf. Figs. 2C and 4A). Also, the bifunctional converter enzyme is assumed to have just a single catalytic site, which is supposed to mimic the fact that the phosphotransferase and phosphatase activities of the sensor kinase in TCSs are also likely to occur on a single catalytic site [17].

The dynamics of this system is described by the set of ordinary differential equations (ODEs)
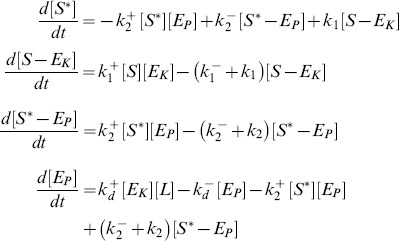
(4)together with the conservation relations for the total concentrations of substrate (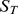), converter enzyme (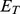) and allosteric effector (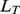)
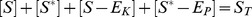
(5)

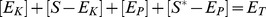
(6)

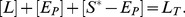
(7)


If the substrate concentration is much larger than that of the converter enzyme (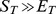), one can neglect the concentrations of the enzyme-substrate complexes (since 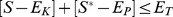 by Eq. 6) in the conservation relation for the substrate (Eq. 5), and the concentration of unmodified substrate can be expressed as
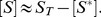
(8)For later comparison, it will be useful to employ the quasi-steady state approximation (QSSA) in order to derive an effective equation for 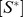. By construction, the QSSA preserves the steady state structure of the underlying system [29] (which is the main focus here) although, for a better description of the transient dynamics, application of the *total* QSSA may be advantageous [30]. To apply the QSSA, it is assumed that, after a short transient period, the enzyme-substrate and the enzyme-effector complexes reach a quasi-steady state, defined by 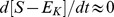, 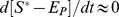 and 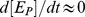, which leads to the algebraic relations
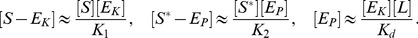
(9)Here, 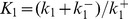 and 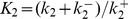 denote Michaelis-Menten constants associated with the kinase and phosphatase activities, respectively, whereas 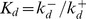 denotes the dissociation constant for the enzyme-effector complex.

Using the QSSA condition 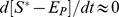, it follows that
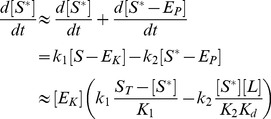
(10)where Eqs. (4), (8) and (9) have been used. In Eq. (10), 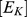 and 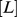 have to be found as functions of 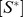 from the conservation relations (Eqs. 6 and 7)
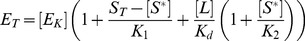
(11)

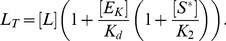
(12)Intuitively, it is clear that if the effector concentration is sufficiently large (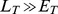) the amount of effector that can be sequestered by the enzyme will be small since 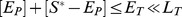. Under this condition the conservation law for the effector (Eqs. 7 and 12) always reduces to 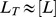 independent of whether the binding affinity of the effector is high (if 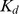 is small) or low (if 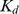 is large). The latter only becomes important when the effector concentration is equal to or smaller than the enzyme concentration (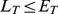), e.g. under effector-limiting conditions. In the following, it will be shown that the type of effective equation, that is obtained from Eqs. (10)–(12), depends on the ratio 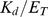 which may be regarded as a relative binding affinity for the enzyme-effector complex.

#### A low-affinity effector generates a graded response

If the relative binding affinity of the enzyme-effector complex is low (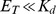) one can neglect the terms associated with the enzyme-effector complexes in Eq. (12) since
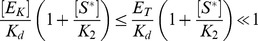
provided that 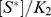 remains sufficiently small. Under this condition, one can use the simplified conservation relation 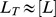 also at low effector concentrations (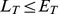), so that 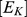 in Eq. (11) can be approximated by
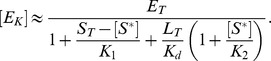
Using this expression together with 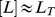 in Eq. (10) yields an effective equation for 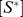 given by
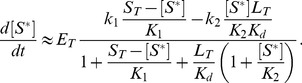
(13)Under steady state conditions (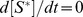) the fraction of modified substrate exhibits a simple hyperbolic dependence on the effector concentration
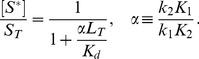
(14)Hence, if reciprocal regulation occurs via a low affinity effector the system exhibits a graded response and ultrasensitivity cannot occur (Fig. 4B, right curves).

#### A high-affinity effector may lead to ultrasensitivity at low effector concentrations

If the relative binding affinity of the enzyme-effector complex is high (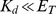) the simplified conservation relation 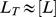 becomes invalid at low effector concentrations (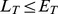). In that case, the combination of Eqs. (11) and (12) leads to a quadratic equation for 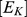, which can be written in the form
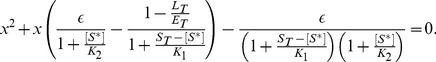
(15)Here, 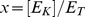 and 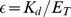 denote the normalized enzyme concentration and the relative binding affinity, respectively. In the limit 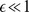, one can neglect the 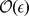 terms in Eq. (15) and obtains, to lowest order, the approximate solution
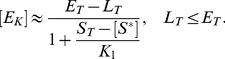
(16)Note that this expression is only valid if the effector concentration is sufficiently small. The second branch of the solution (defined for 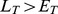) is of 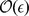 and does not support ultrasensitivity (see *SI Text S1*).

From the expression for 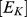 in Eq. (16) it follows that 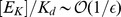, i.e. 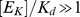. Hence, one may approximate the free effector concentration (as determined by Eq. 12) through
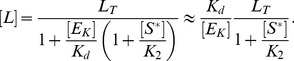
(17)Finally, inserting Eqs. (16) and (17) into Eq. (10) yields the effective equation
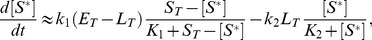
(18)which is essentially the same equation as that derived by Goldbeter and Koshland for covalent modification cycles with two distinct converter enzymes [31]. Hence, if the activities of a bifunctional enzyme with a single catalytic site operate in saturation, so that 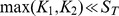, reciprocal regulation of the enzyme's activities by a high-affinity effector may result in zero-order ultrasensitivity similar as predicted by the Goldbeter-Koshland model (Fig. 4B, left curves).

Comparison with the equation for the Goldbeter-Koshland model [31]

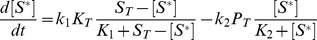
(19)shows that, in Eq. (18), the total kinase concentration (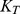) is replaced by 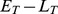 whereas the total phosphatase concentration (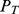) is replaced by 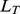. This result has an intuitive interpretation: If the binding affinity of the effector is sufficiently high it can effectively sequester the enzyme into the states with phosphatase activity (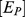 and 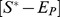) leaving only the enzyme fraction 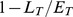 for catalyzing the opposite reaction. In fact, using Eqs. (9), (16) and (17), it is straightforward to show that a high-affinity effector leads to a tight partition of the enzyme states according to
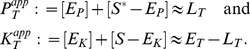
(20)Hence, one may regard 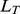 and 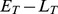 as apparent phosphatase and kinase concentrations, respectively.

From Eqs. (16) and (18), it is also clear that ultrasensitivity becomes observable only at sufficiently low effector concentrations. Specifically, the transition from the ‘on’ (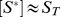) to the ‘off’ (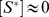) state, defined by 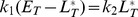, happens at
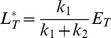
(21)so that the occurrence of ultrasensitivity is limited to the concentration range 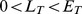, which is consistent with the range of validity of the approximation in Eq. (16).

### Batchelor-Goulian model with reciprocal regulation

The Batchelor-Goulian model is based on the three activities of the sensor kinase shown in Fig. 2B, i.e. it essentially focuses on the signal transduction layer in the general scheme for two-component signaling depicted in Fig. 1. However, within the context of this model it may become difficult to predict the input-output behavior as a function of the input stimulus, especially if the latter affects multiple enzyme activities as observed in the PhoQ/PhoP/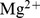 and NRII/NRI/PII systems (Fig. 2A). Guided by these examples the Batchelor-Goulian model will be extended by incorporating a mechanism that accounts for reciprocal regulation of the sensor kinase's autokinase and phosphatase activities by an allosteric effector. Analysis of this model shows that a low-affinity effector may lead to stimulus-dependent concentration robustness whereas a high-affinity effector may generate ultrasensitivity. In the latter case, the underlying mechanism is essentially the same as for covalent modification cycles (cf. Fig. 4).

To implement reciprocal regulation it is assumed (cf. Fig. 2C) that, in the absence of the effector, the free form of the sensor kinase (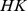) can undergo autophosphorylation and mediates the phosphotransfer to the response regulator (step 1 and 2), but does not exhibit phosphatase activity (step 3). The latter is assumed to be activated through effector-binding (step 4), so that the phosphatase activity is carried by the ligand-bound form of the sensor kinase. Since 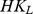 cannot undergo autophosphorylation (and phosphotransfer) binding of the ligand effectively leads to inhibition of the HK's autokinase activity and, concomitantly, activates its phosphatase activity.

The dynamics of the extended model, as shown in Fig. 2C, is described by the five ODEs
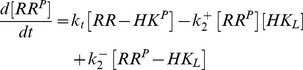
(22)

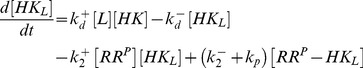
(23)

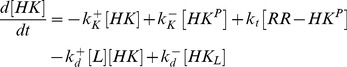
(24)

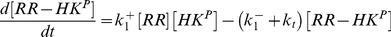
(25)

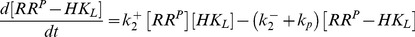
(26)together with the three conservation relations
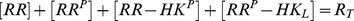
(27)

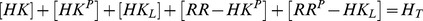
(28)

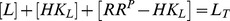
(29)where 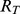, 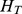 and 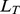 denote the total concentrations of response regulator, histidine kinase and effector, respectively. Measurements in the PhoQ/PhoP and NRII/NRI systems have shown that the ratio between the total concentrations of RR and HK is large (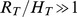) [22], [28] in which case one can use the simplified conservation relation (cf. Eq. 8)
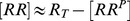
(30)instead of Eq. (27). Similar as in the case of covalent modification cycles (Eqs. 10–12), the steady state behavior of the system, described by Eqs. (22)–(30), depends on the affinity of the allosteric effector (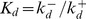) relative to the total enzyme concentration (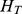).

Note that for the derivation of Eqs. (22)–(30) it has been assumed that signal-sensing and the reactions describing the catalytic activities of the sensor kinase take place in the same compartment (the cytoplasm of the cell). Hence, this model directly applies to cytosolic TCSs, such as the NRII/NRI system, but not to systems with a transmembrane sensor kinase where signal-sensing typically occurs in a different compartment. For example, in the PhoQ/PhoP system the sensor kinase PhoQ responds to changes of the 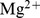 concentration in the periplasm [20]. However, since effector-binding does not involve mass transfer the conditions for the occurrence of concentration robustness and ultrasensitivity are essentially the same (up to a factor accounting for the different compartment volumes) as those which are derived below on the basis of Eqs. (22)–(30) (see *Methods*).

#### A low-affinity effector generates graded responses and stimulus-dependent concentration robustness

If the dissociation constant of the enzyme-effector complex is much larger than the total enzyme concentration (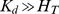) one can replace the conservation relation for the effector (Eq. 29) by 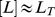, so that the steady state equation for 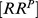 becomes (see *Methods*)
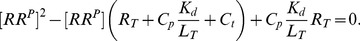
(31)However, this equation coincides with that, derived by Batchelor and Goulian in (Eq. 1), if the parameter 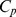 is substituted by the effective parameter
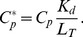
(32)Hence, if the effector exhibits a low affinity ultrasensitivity cannot occur. Instead, Eq. (31) predicts a graded response of 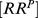 with respect to changes in the effector concentration.

**Figure 5:**
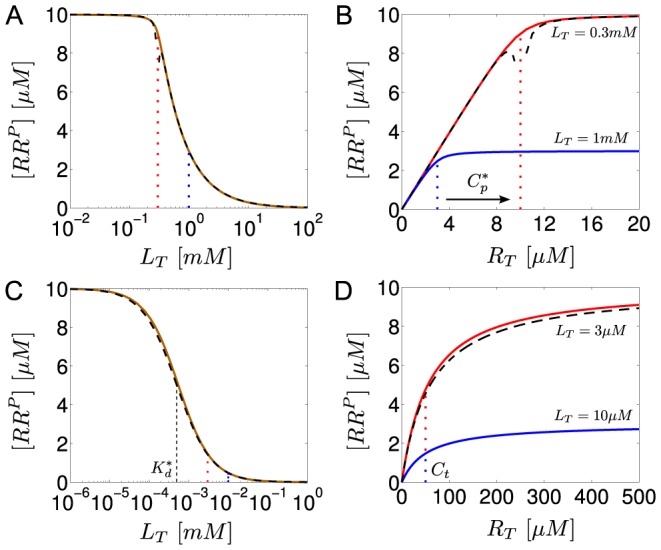
Stimulus-dependent concentration robustness in two-component systems. Steady state response curves according to Eq. (31) for 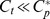 (A and B) and 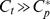 (C and D). (A and C) 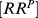 exhibits a graded response as a function of 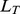. (B and D) 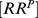 exhibits stimulus-dependent concentration robustness as a function of 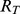. The dotted lines indicate the threshold concentrations beyond which 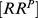 becomes approximately constant. Note that, if 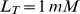 (corresponding to the blue dotted line in A), increasing 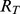 beyond 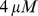 does not lead to a higher phosphorylation level of the response regulator (B), which might explain why autoregulation in TCSs does not necessarily lead to a higher phosphorylation level of the response regulator (cf. Ref. [22]). However, decreasing the effector concentration to 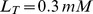 (corresponding to the red dotted line in A) allows 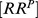 to increase as 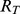 increases. Solid lines were obtained from simulations of the full model (Eqs. 22–29) using the parameters: 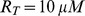, 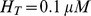, 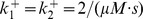, 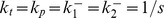, 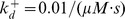, 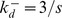 (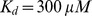, cf. Ref. [46]). (A and B) 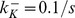, 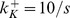 (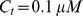, 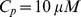) and (C and D) 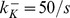, 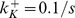 (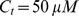, 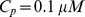). Dashed lines correspond to the approximate solutions in Eq. (33) (A and B) and Eq. (34) (C and D).

To see this more explicitly, it will be useful to consider again the two limiting cases 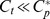 and 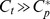, which lead to the approximate solutions (cf. Eqs. 2 and 3)
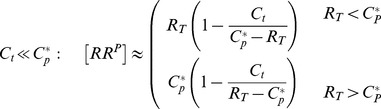
(33)and
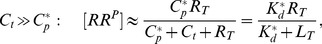
(34)with 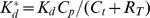. From these expressions, it is apparent that 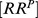 becomes constant at low effector concentrations and decreases as 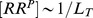 if 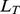 becomes sufficiently large. More precisely, if 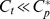 (Eq. 33), 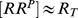 for 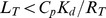 and 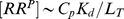 for 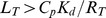 (Fig. 5A). In the opposite case, i.e. if 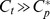 (Eq. 34), the qualitative behavior of 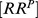 is similar to that described by Eq. (33) although the transition from the state where 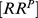 is high (for 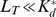) to the state where 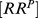 is low (for 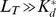) occurs more gradually (Fig. 5C).

More importantly, concentration robustness is now predicted to occur in a stimulus-dependent manner since the maximal phosphorylation level of the RR (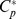) depends on the effector concentration 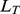 (Eq. 32). However, since only 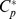 (but not 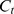) is affected by 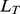 there is a notable difference between the two regimes, described by Eqs. (33) and (34), which may be used to distinguish them experimentally. In the first case, changing the effector concentration will change both, the threshold beyond which concentration robustness occurs and the value of the maximal phosphorylation level (both of which are determined by 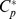) (Fig. 5B). In contrast, when 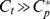 changing 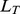 only changes the maximal phosphorylation level while leaving the threshold concentration (which is determined by 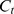) unchanged (Fig. 5D).

#### Stimulus-dependent concentration robustness in the PhoQ/PhoP system

Evidence for stimulus-dependent concentration robustness came from experiments with the PhoQ/PhoP system where Miyashiro and Goulian investigated the effect of genetic autoregulation on the expression level of PhoP-regulated genes at different 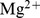 concentrations in the growth medium [22]. At high 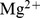 concentrations, they observed almost no effect on PhoP-regulated genes indicating that the concentration of phosphorylated PhoP remained approximately constant under these conditions (despite an expected increase of the total PhoP concentration due to autoregulation of the *phoP* gene). In contrast, under limiting 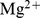 concentrations a substantial increase in the transcript levels of PhoP-regulated genes was detected indicating that the PhoP-P concentration had increased under this condition.

These findings were rationalized based on Eq. (1) by assuming that the parameter 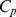, which determines the maximal phosphorylation level of the RR as well as the threshold concentration for reaching this level (Eq. 2), increases as the 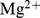 concentration decreases. Interestingly, such an inverse relationship between 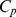 and the effector concentration is readily predicted by the extended model (Eq. 32 and Fig. 5B), where it arises from the assumption that effector binding inhibits the autokinase activity and increases the phosphatase activity of the sensor kinase – in agreement with the observed regulatory effect of 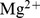 on the activities of PhoQ. Indeed, in the opposite case, if effector binding activated the kinase and inhibited the phosphatase activity, 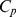 would be proportional to 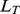.

Although Miyashiro and Goulian did not measure the concentration of PhoP-P directly they observed a gradual (rather than switch-like) increase in the transcript levels of PhoP-regulated genes as the 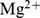 concentration was lowered – in qualitative agreement with the stimulus-response curves depicted in Figs. 5A and 5C. Moreover, measurements using isolated PhoQ sensor domains yielded an apparent dissociation constant for 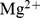 binding of 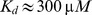 which is much larger than typical intracellular sensor kinase concentrations (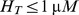) [23], [28]. Together, this supports the view that the PhoQ/PhoP system operates in the low-affinity regime (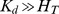) described by Eqs. (33) and (34). Note that this conclusion is not affected by the circumstance that 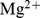 binding occurs in the periplasm. In that case, the low-affinity regime is characterized by the condition 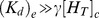 (see *Methods*) where 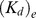 denotes the dissociation constant of the enzyme-effector complex as measured in the periplasm, 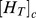 is the cytosolic concentration of the sensor kinase and 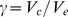 denotes the ratio between the cytosolic and the periplasmic volume.

#### A high-affinity effector may generate ultrasensitivity at low effector concentrations

**Figure 6:**
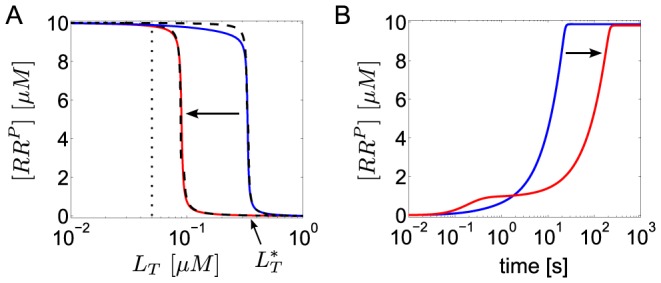
Ultrasensitivity does not require both enzyme activities to be saturated. (A) As the phosphotransferase (PT) activity of the sensor kinase changes from saturation (blue curve) to non-saturation (red curve) the steady state response of 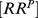 as a function of 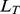 remains ultrasensitive, but the transition point (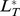), as defined in Eq. (38), is shifted to lower effector concentrations. Blue curve: 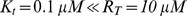, red curve: 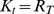. In both cases 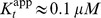. (B) Transient dynamics for 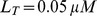 (dotted line in A) indicating that the time-scale for reaching the steady state increases if the PT activity becomes non-saturated. Initial conditions: 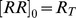, 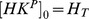, 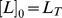, all other concentrations were set to zero. Solid lines were computed from the full model in Eqs. (22)–(29) with the parameters 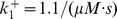, 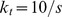, 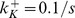, 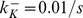 (red curve) and 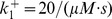, 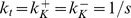 (blue curve). Other parameters: 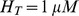, 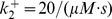, 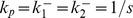, 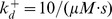, 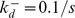, so that 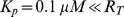 and 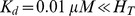. Dashed lines in A correspond to the approximate expression for the stimulus-response curve in Eq. (40).

If the dissociation constant of the enzyme-effector complex is much smaller than the total enzyme concentration (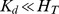) the steady state concentration of 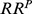 is determined by (see *Methods*)
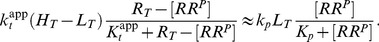
(35)Here, the apparent catalytic rate of the phosphotransferase activity (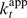) as well as the apparent Michaelis-Menten constant of that activity (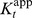) are defined in terms of their intrinsic values (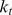 and 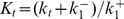) and the kinetic rates (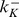 and 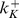) associated with the autophosphorylation activity of the HK through
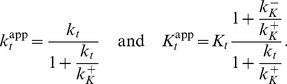
(36)Apparently, Eq. (35) is structurally identical to the steady state equation resulting from Eq. (18), so that the response of 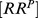 with respect to 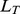 is predicted to become ultrasensitive if
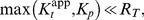
(37)and the transition from the ‘on’ state (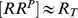) to the ‘off’ state (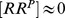) occurs at (cf. Eq. 21)
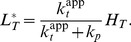
(38)Hence, for TCSs with a bifunctional sensor kinase the occurrence of ultrasensitivity is also restricted to low effector concentrations (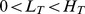) similar to covalent modification cycles with a bifunctional converter enzyme (cf. Eq. 21). However, compared to covalent modification cycles (Eqs. 18 and 19), the occurrence of ultrasensitivity in TCSs with a bifunctional HK only requires the phosphatase activity of the HK to operate in the zero-order regime (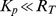). In contrast, the phosphotransferase activity can remain of first order as long as the regulatory factor, which multiplies 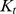 in Eq. (36), is sufficiently small, so that 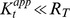 (Eq. 37). Thus, two scenarios are conceivable: First, if the regulatory factor is of order one or larger (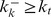) both activities have to operate in saturation (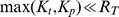) for ultrasensitivity to occur. Second, if the regulatory factor becomes sufficiently small, e.g. when
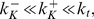
(39)

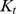
 can become comparable to or larger than 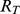 without compromising the system's ability to generate ultrasensitivity (Fig. 6A). However, the condition on the kinetic rate constants in Eq. (39) leads to a shift in the transition point towards lower effector concentrations (Eq. 38) and may, substantially, affect the time scale on which the steady state is reached (Fig. 6B).

Under the condition, stated in Eq. (37), the positive solution of Eq. (35) can be approximated by (see *SI Text S1*)
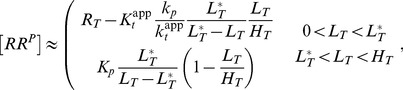
(40)which confirms that there is a sharp transition near the threshold concentration 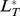, defined in Eq. 38, where the state of the response regular changes from almost full phosphorylation, i.e. 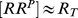 for 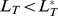, to a nearly unphosphorylated state 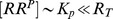 for 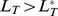 (cf. Fig. 6A).

#### Basal HK phosphatase activity may compromise ultrasensitivity

**Figure 7:**
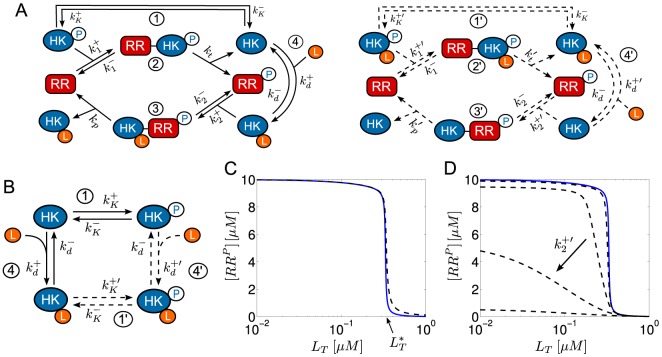
Impact of basal HK activities on the occurrence of ultrasensitivity. (A) Extended Batchelor-Goulian model (cf. Fig. 2C) with basal HK activities (dashed lines): Autophosphorylation (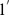), phosphotransfer (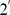) and phosphatase activity (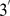). (B) Scheme summarizing the allowed transitions between different enzyme states in the extended model with basal activities. It is assumed that ligand-binding occurs with reduced affinity (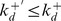) if the sensor kinase has already undergone autophosphorylation (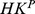). To prevent cycle fluxes under steady state conditions it is required that 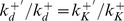. (C) Basal autokinase and phosphotransferase activities hardly affect the response curve. Even if the basal activities are identical to their full activities (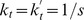, 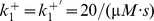, 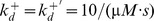, 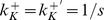) the transition point (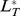) remains almost the same and the steepness of the response curve is only slightly reduced (dashed curve). Basal phosphatase activity is assumed to be zero (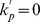, 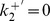). The blue curve is the same as that shown in Fig. 6A where both basal activities are zero. (D) In contrast, upon increasing basal phosphatase activity the steepness of the response curve (ultrasensitivity) becomes substantially reduced. Dashed lines correspond to 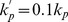 and increasing values of 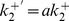 for 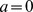 (blue curve), 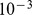, 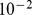, 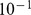, 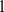. Basal autokinase and phosphotransferase activities are assumed to be zero (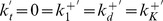). Other parameter values are the same as for the blue curve in Fig. 6A. Simulations were done using Eqs. (64).

In the mechanism depicted in Fig. 2C it has been assumed that only the free form of the sensor kinase (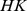) exhibits autokinase activity whereas ligand-binding has been required to activate the phosphatase activity (carried by 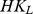). However, it seems reasonable to also consider the more general case where the 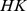 may exhibit some (low) phosphatase activity, even in the absence of effector. Conversely, 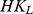 may also undergo autophosphorylation and mediate the phosphotransfer to some extent. To study the impact of such basal activities on the occurrence of ultrasensitivity it has been assumed that 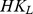 and 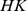 catalyze the same set of reactions (Fig. 7A), but with lower or equal catalytic rate constants for the basal activities (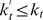, 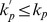 and 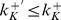). In general, changes in enzyme activity may also result from changes in the binding affinity. To account for such changes the association rate constants were allowed to vary according to 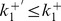 and 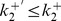 for enzyme-substrate binding and 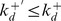 for enzyme-effector binding (Fig. 7B).

As can be seen in Fig. 7C increasing the basal autokinase and phosphotransferase activities of 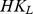, to the extent exhibited by 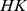, has only a minor effect on the response curve so that the occurrence of ultrasensitivity is not compromised in that case. In contrast, when increasing the basal phosphatase activity of 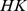 ultrasensitivity gets lost if the affinity between 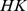 and 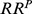 becomes sufficiently large (Fig. 7D). This suggests that, for ultrasensitivity to occur, 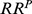 must preferentially bind to 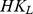 which requires tight regulation of the sensor kinase's phosphatase activity, e.g. through ligand-binding induced conformational changes of the sensor kinase [16].

#### Ultrasensitivity in the NRII/NRI system?

Compared to PhoQ, which is a transmembrane sensor kinase, NRII is located in the cytosol where it controls the expression of nitrogen-regulated genes through reversible phosphorylation of NRI. The PII protein binds to the kinase-domain of NRII which inhibits autophosphorylation, but increases the phosphatase activity of NRII [16], [21]. The components of the NRII/NRI/PII system have been purified and reconstituted *in vitro* [32] making this system amenable to measurements under well-defined conditions without interference from genetic autoregulation or other regulatory systems.

**Figure 8:**
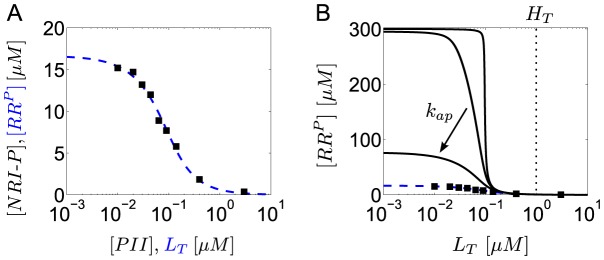
Autophosphatase activity of NRI may compromise ultrasensitivity in the NRII/NRI/PII system. (A) Comparison of experimental data (filled boxes, data taken from Fig. 4A of Ref. [27]) with the steady state response curve calculated from the extended Batchelor-Goulian model in Eqs. (22)–(29) with an extra term ‘
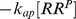
’ added to Eq. (22), which accounts for autodephosphorylation of NRI-P. The blue dashed line represents the best fit obtained for 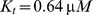, 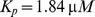, 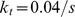 and 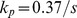. The other parameters were kept fixed: 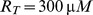, 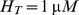, 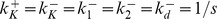, 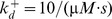 so that 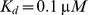 and 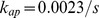 corresponding to a half-life of 5 minutes [27]. (B) As the autodephosphorylation rate constant of NRI-P is lowered (bottom to top: 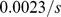, 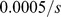, 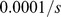, 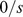) the response curve becomes more and more ultrasensitive (solid lines). Note that ultrasensitivity is restricted to the region 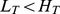 as predicted by Eq. (38). The dashed (blue) lines in (A) and (B) are identical.

In such a setting, Jiang et al. measured the sensitivity of the steady state response of phosphorylated NRI (NRI-P) with respect to PII at different levels of total NRI (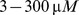) [27]. Half-maximal response occurred at 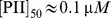 indicating that the 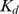 for binding of PII to NRII is (much) smaller than the total enzyme concentration used in the experiments (total 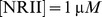). Hence, the two conditions 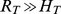 and 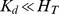, which are required for the applicability of Eq. (35), seem to be fulfilled in the NRII/NRI/PII system, at least under *in vitro* conditions. However, even under saturating substrate levels (total 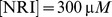) the response curve of NRI-P exhibited only a weak sensitivity with respect to changes in the PII concentration with an effective Hill coefficient of 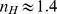 [27] (Fig. 8A).

To explain this weak sensitivity Jiang et al. argued that the activities of NRII might operate in unfavorable kinetic regimes for ultrasensitivity to occur. Specifically, while the kinase/phosphotransferase activities were found to be saturated under the conditions of the experiments the phosphatase activity did not appear to be saturable, which is consistent with the theoretical prediction that ultrasensitivity requires saturation of the phosphatase activity (cf. Eq. 37). However, given that intracellular NRI concentrations presumably lie in the submicromolar range [28] it seems unlikely that the condition 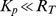 is still violated at total NRI concentrations as large as 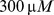. This suggests that either conventional ideas about enzyme saturation are not applicable to the phosphatase activity of NRII (as discussed in Jiang et al. [27]) or that ultrasensitivity is compromised by another mechanism. The latter conclusion is supported by the observation (cf. Fig. 8A) that the maximal phosphorylation level of NRI (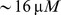) is much lower than the total NRI concentration used in the experiments (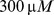) which indicates the presence of a substantial phosphatase activity, even in the absence of effector (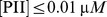).

As indicated by Fig. 7D such an unregulated activity could result from a basal NRII phosphatase activity or, alternatively, from an intrinsic autophosphatase activity of NRI. Since the basal NRII phosphatase activity was found to be quite low [16] the second possibility appears more likely. In fact, compared with that of other response regulators the autophosphatase activity of NRI is comparably high [1] which results in a NRI-P half-life of 5 minutes [27]. To study the impact of NRI-P autodephosphorylation on the occurrence of ultrasensitivity I have added an extra term (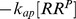) to Eq. (22) and fitted the resulting set of equations to the measurements obtained by Jiang et al. under saturating conditions (Fig. 8A). To this end, only the Michaelis-Menten constants and the catalytic rate constants for (de-)phosphorylation were allowed to vary as these parameters should exhibit the most influence on the steady state response according to Eq. (35). The parameters 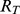, 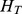, 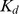 and 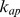 were fixed at their experimental values, whereas the remaining parameters (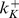, 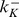, 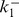, 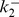 and 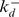) were arbitrarily fixed at 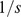 so that they are all large compared to the autodephosphorylation rate constant 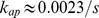. The thus obtained values for the Michaelis-Menten constants (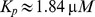 and 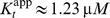) are much lower than the total NRI concentration (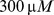) which suggests that the NRII/NRI/PII system operates in a kinetic regime that would, in principle, allow for ultrasensitivity. Hence, by lowering the autophosphatase activity of NRI the fitted response curve should become more and more ultrasensitive which is, indeed, what is observable in Fig. 8B. Together, this supports the view that the intrinsic autophosphatase activity of NRI might play a prominent role for the observed weak sensitivity of the NRII/NRI system under *in vitro* conditions.

## Discussion

In many two-component systems, the phosphorylation level of the response regulator protein is modified by a bifunctional sensor kinase which, apart from exhibiting autokinase and phosphotransferase activity, also catalyzes the dephosphorylation of the response regulator through a phosphatase activity. In the present study, I have argued that the spectrum of potential input-output behaviors of such bifunctional systems does not only comprise graded responses [8]–[10] and concentration robustness [11], [12], but also ultrasensitivity as it is well-known from phosphorylation-dephosphorylation cycles with distinct converter enzymes [31]. To this end, I have proposed and analyzed an extension of the Batchelor-Goulian model [11] which considers the biologically motivated case where the autokinase and phosphatase activities of the sensor kinase are reciprocally regulated by an allosteric effector (Fig. 2).

The analysis of the extended model showed that there exist two operating regimes under steady state conditions depending on the effector affinity: If the affinity is low compared to the total concentration of the sensor kinase (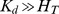) the system produces a graded response to changes in the effector concentration (Eqs. 33 and 34) and exhibits stimulus-dependent concentration robustness, which means that the maximal phosphorylation level of the response regulator does not only depend on kinetic model parameters (as in the original Batchelor-Goulian model), but also on the effector concentration. Consistent with experiments in the PhoQ/PhoP system [22], the extended model predicts an increase in the maximal phosphorylation level as the effector concentration is lowered (Eq. 32). However, if the effector affinity is sufficiently high (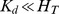) the steady state equation for the extended model (Eq. 35) becomes structurally identical to that for covalent modification cycles with distinct converter enzymes (Eq. 19) so that ultrasensitivity may arise from the zero-order effect [31].

Apart from enzyme saturation due to the zero-order effect, sequestration of a signaling molecule into an inactive complex represents an alternative mechanism for the generation of ultrasensitivity in signal transduction networks [33]–[35]. Often, sequestration involves a reaction of the form
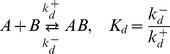
(41)where, by definition, 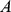 is sequestered by 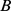 into the complex 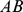. In this sense, regulation of enzyme activities by an allosteric effector may also be regarded as a form of sequestration. In the case of reciprocal regulation shown in Fig. 2C, the enzyme-effector complex (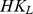) is not catalytically inactive, but rather has a different activity compared to the free form of the enzyme (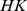). Buchler and Louis have shown that the simple mechanism in Eq. (41) can give rise to ultrasensitivity in the concentrations of 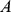 and 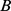 if the stoichiometric binding parameter 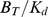 (where 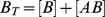) exceeds unity, and the degree of ultrasensitivity increases as 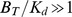 [36]. In the present study, the stoichiometric binding parameter (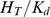) plays a different role for the generation of ultrasensitivity since the condition 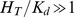 does not guarantee the occurrence of ultrasensitivity *per se*, but only the validity of the reduced model, described by the steady state equation in Eq. (35). To obtain ultrasensitivity within the reduced model, the (apparent) Michaelis-Menten constants for the phosphotransferase and phosphatase activities of the sensor kinase also have to be sufficiently small (Eq. 37), which distinguishes the mechanism, proposed in the present study, from purely sequestration-based mechanisms.

Interestingly, the idea of reciprocal regulation, as a mechanism to generate ultrasensitivity, does not seem to be restricted to two-component systems as the same mechanism may also apply to covalent modification cycles with a bifunctional converter enzyme (Fig. 4A). In both cases, reciprocal regulation may lead to ultrasensitivity if the stoichiometric binding parameters (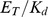 in the case of covalent modification cycles or 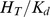 in the case of two-component systems) are sufficiently large. In this case, almost all free effector molecules are bound to the respective enzyme which leads to a tight partition of enzyme states into those with phosphatase activity and those with kinase activity (cf. Eqs. 20 and 52). As a consequence, the system behaves as if phosphorylation and dephosphorylation were catalyzed by independent enzyme subpopulations, which rationalizes why the corresponding steady state equations (Eqs. 18 and 35) are structurally identical to that for covalent modification cycles with distinct converter enzymes (Eq. 19). However, this mechanism only ‘works’ as long as the enzyme is not saturated by the effector, which restricts the occurrence of ultrasensitivity to effector concentrations that are smaller than that of the respective enzyme (Figs. 4B and 6A).

To assess the potential relevance of reciprocal regulation for the occurrence of ultrasensitivity under physiological conditions one has to evaluate to what extent the requirements for its occurrence (substrate excess, a large stoichiometric binding parameter and saturation of the sensor kinase's phosphatase activity) are satisfied in a particular system *in vivo*. Based on measurements in the EnvZ/OmpR, PhoQ/PhoP and PhoR/PhoB systems, it seems that the requirement of substrate excess does not represent a limitation for the occurrence of ultrasensitivity as response regulator proteins are typically much more abundant than their respective sensor proteins [22], [23], [26]. In contrast, estimation of the stoichiometric binding parameter appears more difficult due to the limited knowledge on the range of input signals for a particular sensor kinase and their affinities relative to the total enzyme concentration. In general, histidine kinases may sense different signals (such as ions, metabolites, small peptides or auxiliary proteins) with widely different affinities [3]. Hence, it is conceivable that the same system produces a graded response with respect to a low-affinity effector and an ultrasensitive response with respect to another effector with a high affinity. For example, apart from mediating adaptation to 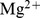-limiting conditions the PhoQ/PhoP system is also involved in the regulation of bacterial virulence. This transcriptional program is initiated by antimicrobial peptides that seem to bind to the same periplasmic site in the sensor domain of PhoQ as 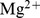, but with a 100-fold higher affinity [37], which could potentially shift the stoichiometric binding parameter into a regime where sigmoidal responses become possible.

The occurrence of ultrasensitivity also requires saturation of the sensor kinase's phosphatase activity which means that the Michaelis-Menten constant, associated with that activity, has to be smaller than the total concentration of the response regulator. Measurements in the EnvZ/OmpR system have shown that the dissociation constant for the EnvZ-OmpR complex is 5-fold smaller than the total OmpR concentration which indicates that enzyme saturation is, in principle, possible under physiological conditions [23]. However, the occurrence of ultrasensitivity can also be compromised by a sufficiently strong, unregulated phosphatase activity which may arise from a basal phosphatase activity of the sensor kinase (Fig. 7D) or from an autophosphatase activity of the response regulator. The latter might explain why the NRII/NRI/PII system exhibits only a weak sensitivity with respect to changes in the effector (PII) concentration (Fig. 8B). Alternatively, it has been speculated that the observed weak sensitivity results from a non-saturable phosphatase activity of NRII [27] which is consistent with the prediction that ultrasensitivity requires the phosphatase activity to operate in the zero-order regime (Eq. 37). On the other hand, it has been shown that single mutations in the dimerization domain of a sensor kinase can substantially affect its interaction strength with cognate and even non-cognate response regulator proteins [26], [38], which suggests that binding affinities between sensor kinases and response regulator proteins are highly evolvable. Hence, it is conceivable that one may employ directed evolution or site-directed mutagenesis to ‘adjust’ these binding affinities in a favorable range for ultrasensitivity to occur. In this sense, the results presented here may also guide the design of synthetic regulatory circuits which aim to implement ultrasensitive response behavior at the level of two-component systems [39].

## Methods

### Steady state analysis of Eqs. (22)–(29)

Under steady state conditions, the right-hand sides of Eqs. (22)–(26) are set to zero so that summation of Eqs. (22) and (26) readily yields
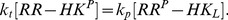
(42)Similarly, summation of Eqs. (23) and (26) leads to the steady state relation
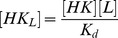
(43)where 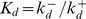 denotes the dissociation constant for the enzyme-effector complex. From Eqs. (24)–(26) together with Eqs. (30) and (43) one obtains the expressions
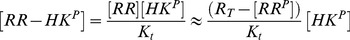
(44)

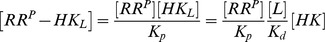


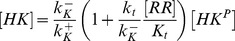


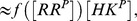
where 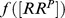 is defined by
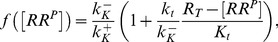
(45)whereas 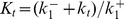 and 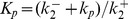 denote Michaelis-Menten constants associated with the phosphotransferase and phosphatase activities of the sensor kinase, respectively.

Using the expressions from Eqs. (43) and (44) in Eq. (42) and in the conservation relations, Eqs. (28) and (29), yields the set of algebraic equations
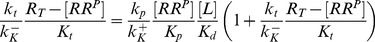
(46)and
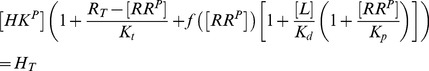
(47)

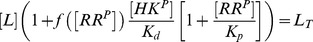
(48)from which the steady state concentrations 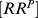, 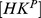 and 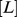 have to be found.

Similar as in the case of a covalent modification cycle with a bifunctional enzyme the type of steady state solution, that is obtained from Eqs. (46)–(48), depends on the affinity of the allosteric effector. If this affinity is low (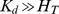) the concentration of free effector is approximately equal to the total effector concentration (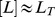). Replacing 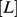 by 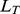 in Eq. (46) readily yields the quadratic equation in Eq. (31) with 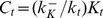 and 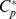 defined in Eq. (32).

In contrast, if the affinity of the effector is sufficiently high (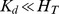) the combination of Eqs. (47) and (48) yields a quadratic equation similar to that in Eq. (15)

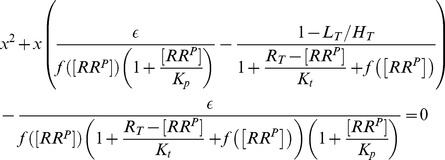
(49)where 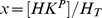 and 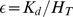 denote the rescaled enzyme concentration and the relative binding affinity, respectively. In the limit 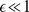, the solution of Eq. (49) can be approximated by [40]

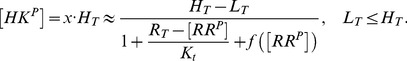
(50)With this approximation the concentration of free effector becomes (cf. Eq. 48)
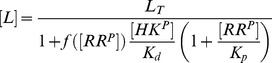
(51)

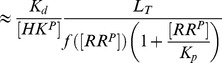


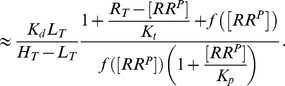
Using this expression for 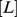 in Eq. (46) yields the equation
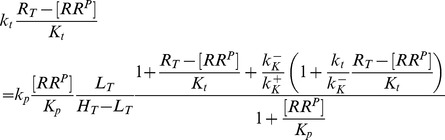
which can be rewritten in the form shown in Eq. (35) of the main text.

Similar to the case of covalent modification cycles it is straightforward to show (using Eqs. 43–45, 50 and 51) that a high-affinity effector leads to a partition of enzyme states according to (cf. Eq. 20)
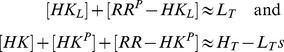
(52)so that 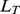 and 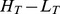 may be regarded as apparent phosphatase and kinase concentrations, respectively.

### Two-compartment model for regulation by an extracellular effector

For TCSs with a transmembrane sensor kinase autophosphorylation, phosphotransfer and dephosphorylation occur in the cytosol whereas signal-sensing typically takes place in the periplasm (for gram-negative bacteria) or directly in the extracellular space (Fig. 1). Hence, a proper model would have to distinguish at least 3 compartments: The cytosol (where the response regulator is located), the plasma membrane (to which the sensor kinase is confined) and the extracellular space (where the effector is located). For gram-negative bacteria one would also have to consider a periplasmic compartment as many sensor kinases seem to respond to signals in the periplasmic rather than directly in the extracellular space [3]. Together, this makes it difficult to propose a generic model for TCSs that are regulated by non-cytosolic effectors which will, therefore, not be attempted here.

Instead, to evaluate the impact of compartmentalization on the conditions for the occurrence of ultrasensitivity and concentration robustness it seems reasonable to consider (as a first approximation) a simplified model where the reactions describing the catalytic activities of the sensor kinase occur in the cytosol (similar as assumed in the original Batchelor-Goulian model) whereas binding of the effector to the regulatory site of the sensor kinase occurs either in the periplasm or in the extracellular space. Because effector-binding does not involve mass transfer between the extracellular space (or the periplasm) and the cytoplasm the equations for such a two-compartment model are essentially the same as those for a single compartment (Eqs. 22–30) if the mass-balance equations are written in terms of average molecule numbers (rather than concentrations). The corresponding ODE system then reads
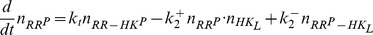
(53)

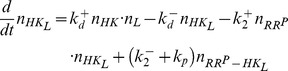


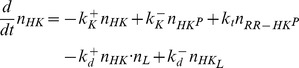


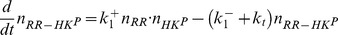


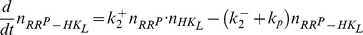
where 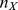 denotes the average amount of species 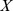 (measured in 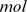). Compared to Eqs. (22)–(26) the second-order rate constants 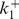, 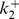 and 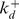 are now measured in units of 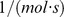, i.e. they are independent of the volume of the compartment in which the corresponding reaction occurs. In contrast, first order rate constants (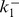, 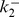, 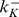, 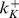, 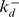, 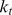 and 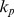) have the same unit (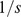) as before. Mass conservation is now expressed in terms of molecule number conservation for the total amount of response regulator (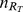), sensor kinase (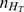) and effector (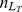) as
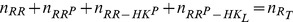
(54)

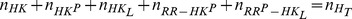


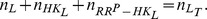
Since the structure of Eqs. (53) and (54) is identical to that of Eqs. (22)–(29) it is clear that the conditions for the occurrence of concentration robustness and ultrasensitivity are identical in both cases if concentration-based quantities are replaced by their respective molar counterparts.

Specifically, ultrasensitivity is predicted to occur if the amount of response regulator is much larger than that of the sensor kinase (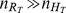) and if the affinity of the effector is sufficiently high. The latter condition is now expressed as
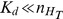
(55)where the dissociation constant 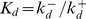 is measured in 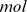. Under these conditions, the steady state amount of phosphorylated response regulator is determined by the analog of Eq. (35)

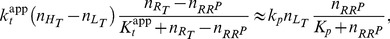
(56)where 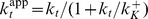 and 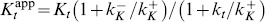 are defined by the same expressions as in Eq. (36). Similar as 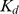, the Michaelis-Menten constants 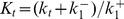 and 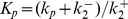 are measured in units of 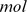. Conversely, if the effector has a low affinity (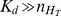) the steady state amount of 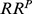 is determined by the analog of Eq. (31)

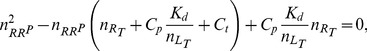
(57)where the rescaled Michaelis-Menten constants 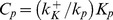 and 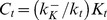 are defined by the same expressions as in Eq. (31).

To analyze the impact of the compartment sizes on the input-output behavior one has to rewrite Eqs. (56) and (57) in terms of concentration-based quantities. For this purpose, the concentrations of the response regulator and that of the sensor kinase
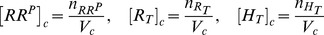
(58)are measured with respect to the cytosolic volume 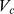, whereas the effector concentration
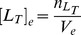
(59)is measured with respect to the extracellular (or periplasmic) volume 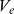. In the case of an extracellular effector, one may think of 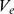 as the effective volume that is accessible to each cell in a population. In general, the effective volume decreases as the number of cells increases, e.g. due to cell growth. However, for the present purpose 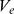 will be taken as a constant parameter. In addition, it is assumed that the extracellular space is a well-mixed compartment so that effector-diffusion can be neglected.

Using the definitions in Eqs. (58) and (59), Eqs. (56) and (57) can be written in the form
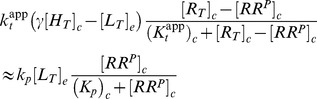
(60)and
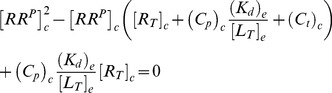
(61)where
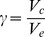
(62)denotes the ratio between the cytosolic volume and that of the extracellular (or periplasmic) space. Also, in Eqs. (60) and (61) the dissociation constant and the Michaelis-Menten constants have been rescaled according to
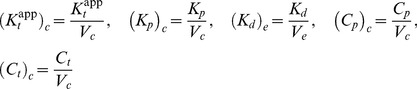
(63)which gives them the conventional unit 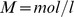. The rescaling is motivated by the fact that, in a concentration-based description of chemical reactions, second-order rate constants have to be proportional to the volume of the compartment in which the corresponding reaction occurs [41], i.e. 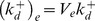, 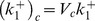 and 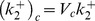 giving them units of 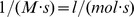.

Similar as Eq. (35), Eq. (60) predicts that ultrasensitivity may occur at low effector concentrations (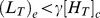) if the affinity of the effector is sufficiently high (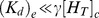). The latter condition follows from Eq. (55) using that 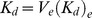 (Eq. 63) and 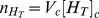 (Eq. 58). Hence, depending on the volume ratio 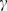 the occurrence of ultrasensitivity may be favored (if 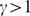) or suppressed (if 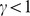) compared to a system that is regulated by a cytosolic effector (for which 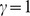). For example, if regulation occurs via a periplasmic effector 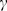 may vary between 1.5 and 4 corresponding to a periplasmic volume fraction of 20–40% of the total cell volume [42]. In contrast, if regulation occurs via an extracellular effector the volume ratio may be substantially smaller than 1 (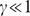) (especially at low cell densities) which would make the condition 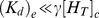 less likely to hold and, therefore, suppress the occurrence of ultrasensitivity.

Interestingly, Eq. (61) does not explicitly depend on the volume ratio. Hence, if reciprocal regulation occurs via a low-affinity extracellular effector (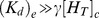) the stimulus-response curves predicted by Eq. (61) are identical with those depicted in Fig. 5 if one replaces 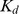 and 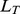 by their extracellular (or periplasmic) counterparts 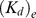 and 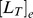, respectively.

### Extended Batchelor-Goulian model with basal HK activities

The response curves in Fig. 7C and 7D have been generated using the following set of equations (the corresponding reaction mechanism is shown in Fig. 7A and 7B)
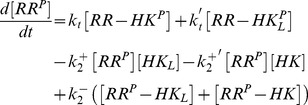
(64)

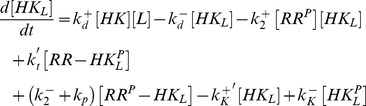


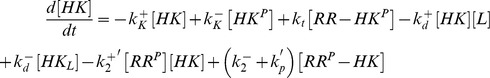


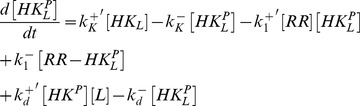


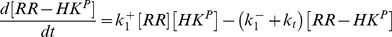


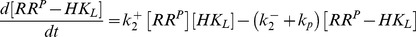


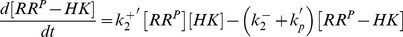


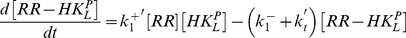
where 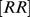, 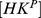 and 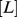 have to be replaced using the conservation relations
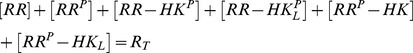


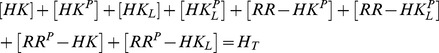


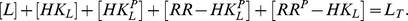



## Supporting Information

Text S1 **Derivations and additional analysis.** This file contains derivations of Eqs. (1)–(3) and (40) as well as the asymptotic analysis of Eq. (15).
(PDF)

## References

[1] Stock AM, Robinson VL, Goudreau PN (2000) Two-component signal transduction. Annu Rev Biochem 69: 183–215. 10966457 10.1146/annurev.biochem.69.1.183

[2] Wuichet K, Cantwell BJ, Zhulin IB (2010) Evolution and phyletic distribution of two-component signal transduction systems. Curr Opin Microbiol 13: 219–225. 20133179 10.1016/j.mib.2009.12.011 PMC3391504

[3] Krell T, Lacal J, Busch A, Silva-Jiménez H, Guazzaroni ME, et al (2010) Bacterial sensor kinases: Diversity in the recognition of environmental signals. Annu Rev Microbiol 64: 539–559. 20825354 10.1146/annurev.micro.112408.134054

[4] Perraud AL, Weiss V, Gross R (1999) Signalling pathways in two-component phosphorelay systems. Trends Microbiol 7: 115–120. 10203840 10.1016/s0966-842x(99)01458-4

[5] Kim JR, Cho KH (2006) The multi-step phosphorelay mechanism of unorthodox two-component systems in *E. coli* realizes ultrasensitivity to stimuli while maintaining robustness to noises. Comput Biol Chem 30: 438–444. 17112785 10.1016/j.compbiolchem.2006.09.004

[6] Potter SL, Armitage JP (2004) Chemotaxis in *Rhodobacter sphaeroides* requires an atypical histidine protein kinase. J Biol Chem 279: 54573–54580. 15485885 10.1074/jbc.M408855200

[7] Amin M, Porter SL, Soyer OS (2013) Split histidine kinases enable ultrasensitivity and bistability in two-component signaling networks. PLoS Comput Biol 9: e1002949. 23505358 10.1371/journal.pcbi.1002949 PMC3591291

[8] Igoshin OA, Alves R, Savageau MA (2008) Hysteretic and graded responses in bacterial two-component signal transduction. Mol Microbiol 68: 1196–1215. 18363790 10.1111/j.1365-2958.2008.06221.x PMC4372536

[9] Alves R, Savageau MA (2003) Comparative analysis of prototype two-component systems with either bifunctional or monofunctional sensors: difference in molecular structure and physiological function. Mol Microbiol 48: 25–51. 12657043 10.1046/j.1365-2958.2003.03344.x

[10] Kremling A, Heermann R, Centler F, Jung K, Gilles ED (2004) Analysis of two-component signal transduction by mathematical modeling using the *KdpD/KdpE* system of *Escherichia coli* . Biosystems 78: 23–37. 15555756 10.1016/j.biosystems.2004.06.003

[11] Batchelor E, Goulian M (2003) Robustness and the cycle of phosphorylation and dephosphorylation in a two-component regulatory system. Proc Natl Acad Sci USA 100: 691–696. 12522261 10.1073/pnas.0234782100 PMC141058

[12] Shinar G, Milo R, Martinez MR, Alon U (2007) Input-output robustness in simple bacterial signaling systems. Proc Natl Acad Sci USA 104: 19931–19935. 18077424 10.1073/pnas.0706792104 PMC2148400

[13] Steuer R, Waldherr S, Sourjik V, Kollmann M (2011) Robust signal processing in living cells. PLoS Comput Biol 7: e1002218. 22215991 10.1371/journal.pcbi.1002218 PMC3219616

[14] Ortega F, Acerenza L, Westerhoff HV, Mas F, Cascante M (2002) Product dependence and bifunctionality compromise the ultrasensitivity of signal transduction cascades. Proc Natl Acad Sci USA 99: 1170–1175. 11830657 10.1073/pnas.022267399 PMC122162

[15] Straube R (2012) Comment on ‘load-induced modulation of signal transduction networks’: Reconciling ultrasensitivity with bifunctionality? Sci Signal 5: lc1. 22215730 10.1126/scisignal.2002699

[16] Jiang P, Atkinson MR, Srisawat C, Sun Q, Ninfa AJ (2000) Functional dissection of the dimerization and enzymatic activities of *Escherichia coli* nitrogen regulator II and their regulation by the PII protein. Biochem 39: 13433–13449. 11063580 10.1021/bi000794u

[17] Stewart RC (2010) Protein histidine kinases: Assembly of active sites and their regulation in signaling pathways. Curr Opin Microbiol 13: 133–141. 20117042 10.1016/j.mib.2009.12.013 PMC2847664

[18] Straube R (2013) Sensitivity and robustness in covalent modification cycles with a bifunctional converter enzyme. Biophys J 105: 1925–1933. 24138868 10.1016/j.bpj.2013.09.010 PMC3797581

[19] Ventura AC, Jiang P, Van Wassenhove L, Del Vecchio D, Merajver SD, et al (2010) Signaling properties of a covalent modification cycle are altered by a downstream target. Proc Natl Acad Sci USA 107: 10032–10037. 20479260 10.1073/pnas.0913815107 PMC2890436

[20] Chamnongpol S, Cromie M, Groisman EA (2003) Mg^2+^ sensing by the Mg^2+^ sensor PhoQ of *Salmonella enterica* . J Mol Biol 325: 795–807. 12507481 10.1016/s0022-2836(02)01268-8

[21] Jiang P, Ninfa AJ (1999) Regulator of autophosphorylation of *Escherichia coli* nitrogen regulator II by the PII signal transduction protein. J Bact 181: 1906–1911. 10074086 10.1128/jb.181.6.1906-1911.1999 PMC93592

[22] Miyashiro T, Goulian M (2008) High stimulus unmasks positive feedback in autoregulated bacterial signaling circuit. Proc Natl Acad Sci USA 105: 17457–17462. 18987315 10.1073/pnas.0807278105 PMC2582279

[23] Cai SJ, Inouye M (2002) EnvZ–OmpR interaction and osmoregulation in *Escherichia coli* . J Biol Chem 277: 24155–24161. 11973328 10.1074/jbc.M110715200

[24] Shinar G, Rabinowitz JD, Alon U (2009) Robustness in glyoxylate bypass regulation. PLoS Comput Biol 5: e1000297. 19266029 10.1371/journal.pcbi.1000297 PMC2645677

[25] Gomez-Uribe C, Verghese GC, Mirny LA (2007) Operating regimes of signaling cycles: Statics, dynamics and noise filtering. PLoS Comput Biol 3: e246. 18159939 10.1371/journal.pcbi.0030246 PMC2230677

[26] Gao R, Stock AM (2013) Probing kinase and phosphatase activities of two-component systems in vivo with concentration-dependent phosphorylation profiling. Proc Natl Acad Sci USA 110: 672–677. 23267085 10.1073/pnas.1214587110 PMC3545780

[27] Jiang P, Ventura AC, Ninfa AJ (2012) Characterization of the reconstituted UTase/UR-PII-NRII-NRI bicyclic signal transduction system that controls the transcription of nitrogen-regulated (Ntr) genes in *Escherichia coli* . Biochem 51: 9045–9057. 23088566 10.1021/bi300575j

[28] Reitzer L (2003) Nitrogen assimilation and global regulation in *Escherichia coli* . Annu Rev Microbiol 57: 155–176. 12730324 10.1146/annurev.micro.57.030502.090820

[29] Segel LA, Slemrod M (1989) The quasi-steady state assumption: A case study in perturbation. SIAM Rev 31: 446–477.

[30] Borghans JAM, de Boer RJ, Segel LA (1996) Extending the quasi-steady state approximation by changing variables. Bull Math Biol 58: 43–63. 8819753 10.1007/BF02458281

[31] Goldbeter A, Koshland DE Jr (1981) An amplified sensitivity arising from covalent modification in biological systems. Proc Natl Acad Sci USA 78: 6840–6844. 6947258 10.1073/pnas.78.11.6840 PMC349147

[32] Jiang P, Peliska JA, Ninfa AJ (1998) Reconstitution of the signal-transduction bicyclic cascade responsible for the regulation of the Ntr gene transcription in *Escherichia coli* . Biochem 37: 12795–12801. 9737856 10.1021/bi9802420

[33] Blüthgen N, Bruggeman FJ, Legewie S, Herzel H, Westerhoff HV, et al (2006) Effects of sequestration on signal transduction cascades. FEBS J 273: 895–906. 16478465 10.1111/j.1742-4658.2006.05105.x

[34] Kim SY, Ferrell JE Jr (2007) Substrate competition as a source of ultrasensitivity in the inactivation of Wee1. Cell 128: 1133–1145. 17382882 10.1016/j.cell.2007.01.039

[35] Martins BM, Swain PS (2013) Ultrasensitivity in phosphorylation-dephosphorylation cycles with little substrate. PLoS Comput Biol 9: e1003175. 23950701 10.1371/journal.pcbi.1003175 PMC3738489

[36] Buchler NE, Louis M (2008) Molecular titration and ultrasensitivity in regulatory networks. J Mol Biol 384: 1106–1119. 18938177 10.1016/j.jmb.2008.09.079

[37] Bader MW, Sanowar S, Daley ME, Schneider AR, Cho US, et al (2005) Recognition of antimicrobial peptides by a bacterial sensor kinase. Cell 122: 461–472. 16096064 10.1016/j.cell.2005.05.030

[38] Siryaporn A, Perchuk BS, Laub MT, Goulian M (2010) Evolving a robust signal transduction pathway from weak cross-talk. Mol Syst Biol 6: 452. 21179024 10.1038/msb.2010.105 PMC3018164

[39] Ninfa AJ (2010) Use of two-component signal transduction systems in the construction of synthetic genetic networks. Curr Opin Microbiol 13: 240–245. 20149718 10.1016/j.mib.2010.01.003 PMC3547608

[40] Straube R, Conradi C (2013) Reciprocal enzyme regulation as a source of bistability in covalent modification cycles. J Theor Biol 330: 56–74. 23583955 10.1016/j.jtbi.2013.04.002

[41] Gillespie DT (1977) Exact stochastic simulation of coupled chemical reactions. J Phys Chem 81: 2340–2361.

[42] Stock JB, Rauch B, Roseman S (1977) Periplasmic space in *Salmonella typhimurium* and *Escherichia coli* . J Biol Chem 252: 7850–7861. 334768

[43] Swem LR, Gong X, Yu CA, Bauer CA (2006) Identification of a ubiquinone-binding site that affects autophosphorylation of the sensor kinase RegB. J Biol Chem 281: 6768–6775. 16407278 10.1074/jbc.M509687200 PMC2776112

[44] Timmen M, Bassler BL, Jung K (2006) Al-1 inuences the kinase activity but not the phosphatase activity of LuxN of *Vibrio harveyi* . J Biol Chem 34: 24398–24404. 16807235 10.1074/jbc.M604108200

[45] Brandon L, Dorus S, Epstein W, Altendorf K, Jung K (2000) Modulation of KdpD phosphatase implicated in the physiological expression of the Kdp ATPase of *Escherichia coli* . Mol Microbiol 38: 1086–1092. 11123681 10.1046/j.1365-2958.2000.02219.x

[46] Lesley JA, Waldburger CD (2001) Comparison of the *Pseudomonas aeruginosa* and *Escherichia coli* PhoQ sensor domains. J Biol Chem 276: 30827–30833. 11404360 10.1074/jbc.M104262200

