# Supplementary material for: Reciprocal Regulation as a Source of Ultrasensitivity in Two-Component Systems with a Bifunctional Sensor Kinase
Source: PLoS Comput Biol. 2014 May 8;10(5):e1003614. doi: 10.1371/journal.pcbi.1003614 (PMC4014401; doi:10.1371/journal.pcbi.1003614)
Supplement: Text S1 — Derivations and additional analysis. This file contains derivations of Eqs. (1)–(3) and (40) as well as the asymptotic analysis of Eq. (15). (PDF) [file pcbi.1003614.s001.pdf]

Supporting Information: S1 Text
for "Reciprocal regulation as a source of ultrasensitivity in two-component systems with a bifunctional sensor kinase"

Ronny Straube[1]

Analysis and Redesign of Biological Networks Group, Max Planck Institute for Dynamics of Complex Technical Systems, Sandtorstrasse 1, D-39106 Magdeburg, Germany

[1]email: rstraube@mpi-magdeburg.mpg.de

Contents

S1 Text **S2**
S1.1 Derivation of Eq. (1) S2
S1.2 Derivation of Eqs. (2) and (3) S4
S1.3 Asymptotic analysis of Eq. (15) S6
S1.4 Derivation of Eq. (40) S7

S1 Text

S1.1 Derivation of Eq. (1)

The Batchelor-Goulian model, as depicted in Fig. 2B, is described by the system of ordinary differential equations

$$\begin{aligned}
\frac{d\left[RR^P\right]}{dt} &= k_t\left[RR\text{-}HK^P\right] + k_2^-\left[RR^P\text{-}HK\right] \\
&\quad -k_2^+\left[RR^P\right][HK] && \text{(S1)}\\
\frac{d[HK]}{dt} &= -k_K^+[HK] + k_K^-\left[HK^P\right] + k_t\left[RR\text{-}HK^P\right] \\
&\quad -k_2^+\left[RR^P\right][HK] + \left(k_2^- + k_p\right)\left[RR^P\text{-}HK\right] && \text{(S2)}\\
\frac{d\left[RR\text{-}HK^P\right]}{dt} &= k_1^+[RR]\left[HK^P\right] - \left(k_1^- + k_t\right)\left[RR\text{-}HK^P\right] && \text{(S3)}\\
\frac{d\left[RR^P\text{-}HK\right]}{dt} &= k_2^+\left[RR^P\right][HK] - \left(k_2^- + k_p\right)\left[RR^P\text{-}HK\right] && \text{(S4)}
\end{aligned}$$

together with the conservation relations for the response regulator (R_T) and the sensor kinase (H_T)

$$\begin{aligned}
[RR] + \left[RR^P\right] + \left[RR\text{-}HK^P\right] + \left[RR^P\text{-}HK\right] &= R_T && \text{(S5)}\\
[HK] + \left[HK^P\right] + \left[RR\text{-}HK^P\right] + \left[RR^P\text{-}HK\right] &= H_T. && \text{(S6)}
\end{aligned}$$

If the response regulator is in excess ($R_T \gg H_T$) one can neglect the concentrations for the enzyme-substrate complexes in Eq. (S5) (since $\left[RR\text{-}HK^P\right] + \left[RR^P\text{-}HK\right] \leq H_T \ll R_T$) so that the conservation relation for the response regulator (Eq. S5) simplifies to

$$[RR] \approx R_T - \left[RR^P\right]. \quad \text{(S7)}$$

Under steady state conditions the left-hand sides in Eqs. (S1) - (S4) can be set to zero. For Eqs. (S3) and (S4) this leads to

$$\begin{aligned}
\left[RR\text{-}HK^P\right] &= \frac{[RR]\left[HK^P\right]}{K_t} \approx \frac{\left(R_T - \left[RR^P\right]\right)\left[HK^P\right]}{K_t} && \text{(S8)}\\
\left[RR^P\text{-}HK\right] &= \frac{\left[RR^P\right][HK]}{K_p} && \text{(S9)}
\end{aligned}$$

where the Michaelis-Menten constants are defined by

$$K_t = \frac{k_t + k_1^-}{k_1^+} \quad \text{and} \quad K_p = \frac{k_p + k_2^-}{k_2^+}.$$

Addition of Eqs. (S2) and (S4) yields

$$\begin{aligned} [HK] &= \frac{k_K^-}{k_K^+}\left[HK^P\right] + \frac{k_t}{k_K^+}\left[RR\text{-}HK^P\right] \\ &= \frac{k_K^-}{k_K^+}\left(1 + \frac{k_t}{k_K^-}\frac{[RR]}{K_t}\right)\left[HK^P\right] \\ &\approx \frac{k_K^-}{k_K^+}\left(1 + \frac{k_t}{k_K^-}\frac{R_T - \left[RR^P\right]}{K_t}\right)\left[HK^P\right]. \end{aligned} \tag{S10}$$

Similarly, addition of Eqs. (S1) and (S4) yields

$$k_t\left[RR\text{-}HK^P\right] = k_p\left[RR^P\text{-}HK\right]$$

and, after substituting the expressions in Eqs. (S8) and (S9),

$$k_t\frac{\left(R_T - \left[RR^P\right]\right)\left[HK^P\right]}{K_t} = k_p\frac{\left[RR^P\right][HK]}{K_p}.$$

Finally, replacing $[HK]$ by the expression in Eq. (S10) the factor $\left[HK^P\right]$ cancels on both sides of the equation and one obtains

$$k_t\frac{R_T - \left[RR^P\right]}{K_t} = k_p\frac{\left[RR^P\right]}{K_p}\frac{k_K^-}{k_K^+}\left(1 + \frac{k_t}{k_K^-}\frac{R_T - \left[RR^P\right]}{K_t}\right).$$

By defining the rescaled Michaelis-Menten constants C_p and C_t through $C_p = \left(k_K^+/k_p\right)K_p$ and $C_t = \left(k_K^-/k_t\right)K_t$ this equation can be written in the form

$$C_p\left(R_T - \left[RR^P\right]\right) = \left[RR^P\right]\left(C_t + R_T - \left[RR^P\right]\right)$$

which directly leads to Eq. (1) of the main text.

S1.2 Derivation of Eqs. (2) and (3)

Within the Batchelor-Goulian model (Fig. 2B) the steady state concentration of the phosphorylated form of the response regulator (RR^P) is determined by the quadratic equation (Eq. 1 of the main text)

$$[RR^P]^2 - [RR^P]\,(R_T + C_p + C_t) + C_p R_T = 0 \tag{S11}$$

which is valid if the concentration of the response regulator is much higher than that of the sensor kinase ($R_T \gg H_T$). In the following, approximate solutions of this equation are derived, which are either valid in the limit $C_t \ll C_p$ or in the limit $C_p \ll C_t$.

In the first case ($C_t \ll C_p$), it is advantageous to introduce the following dimensionless quantities

$$x = \frac{[RR^P]}{R_T}, \quad \bar{C}_p = \frac{C_p}{R_T} \sim \mathcal{O}(1), \quad \varepsilon = \frac{C_t}{C_p} \ll 1 \tag{S12}$$

through which Eq. (S11) becomes

$$x^2 - x\left(1 + \bar{C}_p\,(1+\varepsilon)\right) + \bar{C}_p = 0. \tag{S13}$$

The solution of this equation is sought in the form

$$x = x_0 + \varepsilon x_1 + \mathcal{O}(\varepsilon^2). \tag{S14}$$

Inserting this expansion into Eq. (S13) and equating terms of equal order of magnitude leads, to lowest order ($\varepsilon = 0$), to the quadratic equation

$$x_0^2 - x_0\left(1 + \bar{C}_p\right) + \bar{C}_p = 0\,,$$

which has the two solutions

$$x_0 = \frac{1+\bar{C}_p}{2} \pm \left|\frac{1-\bar{C}_p}{2}\right|. \tag{S15}$$

By definition, $x = [RR^P]/R_T$ must remain within the range $0 \le x \le 1$ which requires to choose the '$-$' sign in Eq. (S15). Hence, the physiologically reasonable solution is given by

$$x_0 = \frac{1+\bar{C}_p}{2} - \left|\frac{1-\bar{C}_p}{2}\right| = \begin{cases} 1\,, & \bar{C}_p > 1 \\ \bar{C}_p\,, & \bar{C}_p < 1 \end{cases}. \tag{S16}$$

The $\mathcal{O}(\varepsilon)$-equation

$$2x_0x_1 - x_0\bar{C}_p - x_1\left(1+\bar{C}_p\right) = 0$$

has the solution

$$x_1 = \begin{cases} -\frac{\bar{C}_p}{\bar{C}_p-1} & \bar{C}_p > 1 \\ -\frac{\bar{C}_p^2}{1-\bar{C}_p} & \bar{C}_p < 1 \end{cases} . \tag{S17}$$

Combining Eqs. (S16) and (S17) shows that the solution of Eq. (S13) can be approximated (up to terms of $\mathcal{O}(\varepsilon^2)$) by

$$x \approx \begin{cases} 1-\varepsilon\frac{\bar{C}_p}{\bar{C}_p-1} & \bar{C}_p > 1 \\ \bar{C}_p\left(1-\varepsilon\frac{\bar{C}_p}{1-\bar{C}_p}\right) & \bar{C}_p < 1 \end{cases} .$$

Switching back to original variables using Eq. (S12) yields Eq. (2) of the main text.

In the limit $C_t \gg C_p$, one may rewrite the exact solution of Eq. (S11)

$$[RR^P] = \frac{R_T + C_p + C_t}{2}\left(1 - \sqrt{1 - 4\frac{C_pR_T}{\left(R_T + C_p + C_t\right)^2}}\right)$$

in terms of the dimensionless quantities

$$x = \frac{[RR^P]}{R_T}, \quad \bar{C}_t = \frac{C_t}{R_T}, \quad \varepsilon = \frac{C_p}{C_t} \ll 1\,, \tag{S18}$$

which yields

$$x = \frac{1+\bar{C}_t\,(1+\varepsilon)}{2}\left(1 - \sqrt{1 - \varepsilon\frac{4\bar{C}_t}{\left(1+\bar{C}_t(1+\varepsilon)\right)^2}}\right) . \tag{S19}$$

Using that $\sqrt{1-\varepsilon x} \approx 1 - \varepsilon x/2$ for $\varepsilon \ll 1$, one may then expand the square root in Eq. (S19) which leads to

$$x \approx \frac{\varepsilon\bar{C}_t}{1+\bar{C}_t(1+\varepsilon)} \approx \frac{\varepsilon\bar{C}_t}{1+\bar{C}_t}\,,$$

where the first approximation (with the ε-term in the denominator) has been used in Eq. (3) of the main text (after switching back to original variables using Eq. S18).

S1.3 Asymptotic analysis of Eq. (15)

To find an approximate solution of Eq. (15) of the main text one may employ the same expansion as in Eq. (S14). Inserting this expansion into Eq. (15) leads, to lowest order ($\varepsilon = 0$), to the quadratic equation

$$x_0^2 - x_0 \frac{1 - \frac{L_T}{E_T}}{1 + \frac{S_T - [S^*]}{K_1}} = 0\,, \tag{S20}$$

which has the two solutions

$$x_0^{(1)} = 0 \quad \text{and} \quad x_0^{(2)} = \frac{1 - \frac{L_T}{E_T}}{1 + \frac{S_T - [S^*]}{K_1}}\,. \tag{S21}$$

By definition, $x = [E_K]/E_T \geq 0$ must not become negative so that $x_0^{(2)}$ is only defined for $L_T \leq E_T$. The range of definition for $x_0^{(1)}$ is determined by the $\mathcal{O}(\varepsilon)$-equation which has the form

$$2x_0 x_1 + \frac{x_0}{1 + \frac{[S^*]}{K_2}} - x_1 \frac{1 - \frac{L_T}{E_T}}{1 + \frac{S_T - [S^*]}{K_1}} - \frac{1}{\left(1 + \frac{[S^*]}{K_2}\right)\left(1 + \frac{S_T - [S^*]}{K_1}\right)} = 0\,. \tag{S22}$$

Its solutions are given by

$$x_1^{(1)} = \frac{1}{1 + \frac{[S^*]}{K_2}} \frac{1}{\frac{L_T}{E_T} - 1} \quad \text{and} \quad x_1^{(2)} = \frac{1}{1 + \frac{[S^*]}{K_2}} \frac{\frac{L_T}{E_T}}{1 - \frac{L_T}{E_T}}\,, \tag{S23}$$

where $x_1^{(1)}$ and $x_1^{(2)}$ are obtained by inserting $x_0^{(1)}$ and $x_0^{(2)}$ into Eq. (S22), respectively. From the expression for $x_1^{(1)}$ in Eq. (S23) it is apparent that this solution (and thus $x_0^{(1)}$) is defined for $L_T > E_T$, so that $x_1^{(1)} > 0$.

By combining Eqs. (S21) and (S23), the solution of Eq. (15) can be approximated (up to terms of $\mathcal{O}(\varepsilon^2)$) by

$$x \approx \begin{cases} \frac{1-\frac{L_T}{E_T}}{1+\frac{S_T-[S^*]}{K_1}} + \frac{\varepsilon}{1+\frac{[S^*]}{K_2}} \frac{\frac{L_T}{E_T}}{1-\frac{L_T}{E_T}}\,, & L_T < E_T \\ \frac{\varepsilon}{1+\frac{[S^*]}{K_2}} \frac{1}{\frac{L_T}{E_T}-1}\,, & L_T > E_T \end{cases}\,. \tag{S24}$$

The approximate expression for $[E_K]$, as used in Eq. (16) of the main text, is obtained from the $\mathcal{O}(1)$-term ($L_T < E_T$) by the replacement $x = [E_K]/E_T$.

The same replacement, together with $\varepsilon = K_d/E_T$, yields the following expression for $[E_K]$ in the region $L_T > E_T$

$$[E_K] \approx \frac{K_d}{1+\frac{[S^*]}{K_2}} \frac{E_T}{L_T - E_T}\,. \tag{S25}$$

Using this expression in the conservation relation for $[L]$ in Eq. (12) of the main text shows that, in the region $L_T > E_T$, the free effector concentration is given by $[L] \approx L_T - E_T$. This result is intuitively clear: For $L_T \geq E_T$ the converter enzyme becomes saturated by effector molecules since $K_d \ll E_T$ (by assumption). Hence, increasing L_T beyond E_T will mainly increase the free effector concentration. Using $[L] \approx L_T - E_T$ in Eq. (10) of the main text shows that, in the region $L_T > E_T$, ultrasensitivity cannot occur since $[S^*]$ exhibits a simple hyperbolic dependence on L_T which is given by

$$[S^*] \approx \frac{S_T}{1+\frac{k_2}{k_1}\frac{K_1}{K_2}\frac{L_T-E_T}{K_d}}\,, \quad L_T > E_T\,. \tag{S26}$$

S1.4 Derivation of Eq. (40)

Eq. (35) of the main text can be written as a quadratic equation

$$[RR^P]^2 - [RR^P]\left(R_T + \frac{\alpha K_p + K_t^{\mathrm{app}}}{1-\alpha}\right) + \frac{\alpha K_p R_T}{1-\alpha} = 0 \tag{S27}$$

where α is given by

$$\alpha = \frac{k_t^{\mathrm{app}}}{k_p}\frac{H_T - L_T}{L_T} > 0\,. \tag{S28}$$

To derive an approximate expression for the solution of Eq. (S27), which is valid under the condition $\max(K_t^{\mathrm{app}}, K_p) \ll R_T$ (Eq. 37), it is advantageous to introduce the dimensionless quantities

$$x = \frac{[RR^P]}{R_T}, \quad K = \frac{K_t^{\mathrm{app}}}{K_p} \sim \mathcal{O}(1), \quad \varepsilon = \frac{K_p}{R_T} \ll 1 \tag{S29}$$

through which Eq. (S27) becomes

$$x^2 - x\left(1 + \varepsilon\frac{K+\alpha}{1-\alpha}\right) + \frac{\varepsilon\alpha}{1-\alpha} = 0\,. \tag{S30}$$

Eq. (S30) has been analyzed previously for the special case $K = 1$ [1]. Following the same steps as in Ref. [1] it is straightforward to show that, in the limit $\varepsilon \ll 1$, the solution of Eq. (S30) can be approximated (up to terms of $\mathcal{O}(\varepsilon^2)$) by

$$x \approx \begin{cases} 1 - \varepsilon \frac{K}{\alpha - 1}\,, & \alpha > 1 \\ \varepsilon \frac{\alpha}{1-\alpha}\,, & \alpha < 1 \end{cases} . \tag{S31}$$

Using the definition of α in Eq. (S28) the conditions $\alpha > 1$ ($\alpha < 1$) become $L_T < L_T^*$ ($L_T > L_T^*$) where L_T^* is defined by (cf. Eq. 38 of the main text)

$$L_T^* = \frac{k_t^{\text{app}}}{k_t^{\text{app}} + k_p} H_T\,. \tag{S32}$$

Finally, Eq. (40) in the main text is obtained from Eq. (S31) by using Eqs. (S28) and (S29), together with the definition of L_T^* in Eq. (S32).

References

[1] Straube R. 2013. Sensitivity and robustness in covalent modification cycles with a bifunctional converter enzyme. *Biophys. J* 105: 1925–1933.
